# Single-Cell Landscape Change in Cervical Epithelial Cells and Microenvironment During the Transformation from CINIII to Cervical Squamous Cell Carcinoma

**DOI:** 10.3390/cancers18101674

**Published:** 2026-05-21

**Authors:** Yaomei Ma, Su Zhang, Bei Liu, Yibo Liu, Yuchao He, Wenchen Gong, Wenshuai Chen, Lisha Qi, Ke Wang, Hua Guo

**Affiliations:** 1 Department of Gynecologic Oncology, Key Laboratory of Cancer Prevention and Therapy, National Clinical Research Center for Cancer, Tianjin Medical University Cancer Institute and Hospital, Tianjin 300060, China; mym905@sina.com (Y.M.); zhangsu@tmu.edu.cn (S.Z.); 2 Tianjin Key Laboratory of Digestive Cancer, National Clinical Research Center for Cancer, Tianjin Medical University Cancer Institute and Hospital, Tianjin 300060, China; liubei227@tmu.edu.cn (B.L.); heyuchao7557@163.com (Y.H.); gongwenchen@tmu.edu.cn (W.G.); wenshuaichen@163.com (W.C.); lqi01@tmu.edu.cn (L.Q.); 3 Tianjin’s Clinical Research Center for Cancer, National Clinical Research Center for Cancer, Tianjin Medical University Cancer Institute and Hospital, Tianjin 300060, China; 4 State Key Laboratory of Druggability Evaluation and Systematic Translational Medicine, National Clinical Research Center for Cancer, Tianjin Medical University Cancer Institute and Hospital, Tianjin 300060, China; 5 Department of Tumor Cell Biology, Key Laboratory of Cancer Prevention and Therapy, National Clinical Research Center for Cancer, Tianjin Medical University Cancer Institute and Hospital, Tianjin 300060, China; 6 Department of Computer Science and Technology, Beijing Jiaotong University, Beijing 100044, China; 22722091@bjtu.edu.cn; 7 Department of Pathology, Key Laboratory of Cancer Prevention and Therapy, National Clinical Research Center for Cancer, Tianjin Medical University Cancer Institute and Hospital, Tianjin 300060, China

**Keywords:** CINIII, CSCC, single cell sequencing, cervical epithelial cells, tumor microenvironment

## Abstract

**Background:** The rising incidence of CINIII, particularly in younger patients, has highlighted limitations of current surgical treatments, which can affect fertility and carry recurrence risks. This underscores an urgent clinical need for non-invasive therapies. Our study addresses this by investigating the cellular and molecular changes during CINIII progression to cervical squamous cell carcinoma (CSCC). **Methods:** We employed single-cell sequencing to meticulously analyze cell types and molecular mechanisms within cervical epithelial cells and their microenvironment throughout the CINIII-to-CSCC transition. **Results:** Key findings include the identification of Sox2 and its signaling pathway as markers for specific cervical stem cells (CCSCs) during malignant transformation. In the microenvironment, upregulated VWF, MMP2, and HTRA1 were observed in vascular endothelial cells, while TXNIP+ and ARL4D+ fibroblasts underwent transformation into myofibroblasts. Immune cell proportions notably increased, particularly macrophages, T cells, B cells, NK cells, mast cells, and neutrophils, contrasting with a decrease in non-immune cells. Furthermore, interaction analysis revealed that communication between macrophages and cervical epithelial cells was the most prominent. **Conclusions:** This research comprehensively details the complex cellular and molecular remodeling inherent in CINIII progression. By pinpointing specific pathways and cell populations, our findings establish a crucial framework for developing innovative, non-invasive drug therapies to delay disease progression and ultimately improve long-term reproductive outcomes for patients.

## Simple Summary

Driven by the increasing incidence of CINIII in younger patients and limitations of surgical treatments, this study addresses the critical need for non-invasive CINIII therapies. Our goal was to uncover cellular and molecular changes during CINIII’s progression to cervical squamous cell carcinoma using single-cell sequencing. We discovered that Sox2 signaling marks cervical cancer stem cells, while key genes, including VWF, MMP2, and HTRA1, are upregulated in cancer-associated endothelial cells, and fibroblasts undergo transformation into myofibroblasts. The immune microenvironment also shifts, notably with increased macrophages. These findings provide crucial insights into disease progression, offering the research community potential targets for drug-based, non-invasive treatments to delay CINIII progression and preserve fertility.

## 1. Introduction

Cervical cancer (CC) ranks as the fourth leading cause of death among women globally, with approximately 660,552 new cases and 350,021 deaths in 2022 [1]. Cervical squamous cell carcinoma (CSCC) constitutes the overwhelming majority of CC cases, making up as much as 70% [2]. The key risk factors implicated in the development of CSCC include high-risk human papillomavirus (hrHPV) infection, age, smoking, childbirth, the use of oral contraceptives, and dietary patterns. Among these diverse risk factors, persistent hrHPV infection serves as the principal cause of CSCC incidence [3]. CSCC originates from normal cervical epithelium. In the early stages, hrHPV-associated CSCC is asymptomatic, as hrHPV infection is latent. It continuously triggers the development of low- to high-grade cervical intraepithelial neoplasia (CINI to CINII and CINIII), eventually leading to CSCC. Once hrHPV infects the cervical epithelium, it induces alterations in the host genome. On one hand, this leads to the silencing of multiple tumor suppressor genes; on the other hand, it induces abnormal expression of various tumor-promoting factors [4]. To date, 216 HPV subtypes have been identified and categorized into high-risk and low-risk types. Among high-risk subtypes, particularly type 16 and type 18 [5], are the primary instigators of cervical cancer transformation, with type 16 having the highest probability of malignant transformation. Thus, hrHPV16-positive specimens are commonly selected for CSCC-related studies [6]. In China, the national cervical cancer screening program primarily employs a strategy of primary hrHPV testing for average-risk women, with genotyping for HPV 16/18 and pooled testing for 12 other high-risk types. For women positive for the other 12 types, liquid-based cytology is used as a triage test to determine the need for colposcopy referral. Despite the availability of HPV testing and cervical cancer screening programs, the incidence and mortality of CSCC in China have not witnessed a significant decline, mainly due to a low screening coverage rate of merely 20–30% among the target population [7]. Suboptimal HPV vaccination coverage further complicates prevention. The national program offers bivalent, quadrivalent, and nonavalent vaccines, primarily targeting adolescent girls. Current coverage remains low (estimated below 50%), and vaccination is not yet routinely recommended for males in China [8]. Despite screening and vaccination efforts, cervical cancer incidence in China tripled from 2000 to 2020, with marked age and urban–rural disparities, while CINIII is increasingly diagnosed in young, unmarried or childless women [9].

A study conducted by the Cancer Hospital of the Chinese Academy of Medical Sciences in 2019 and published in the Lancet Public Health journal indicated that, under a scenario of constant budget scenario, China could completely eliminate cervical cancer by 2070 with the adoption of optimal prevention and control strategies. Even with an unrestricted budget increase along with optimal prevention and control measures, cervical cancer would be eradicated in urban areas of China by 2057 and in rural areas by 2060, respectively [10]. Regular health examinations targeting CSCC play a crucial role in the early detection and treatment of this malignancy. The progression of CINIII varies among patients. A small proportion (20–30%) of CINIII cases will progress to CSCC, while the majority will remain stable for several years, and in a few patients, the condition may even regress to CINI or revert to normal [11]. However, the underlying mechanism remains ambiguous [12]. The relatively low vaccination rate of the cervical cancer vaccine has contributed to a continuous upward trend in the incidence of cervical precancerous lesions over the past three decades. For CINIII, all major treatment guidelines advocate partial cervicectomy [11]. This single-treatment modality requires patients to have part of their cervix removed, which can lead to complications such as preterm labor, prenatal infection, and an elevated fetal mortality rate. Moreover, the cervix cannot regenerate after resection, and it cannot be excluded that cervical lesions may recur following future HPV infection [13]. Currently, there is a growing number of unmarried and/or childless young women diagnosed with CINIII, thus heightening the demand for non-invasive treatment options.

Single-cell sequencing technology enables the sequencing of the genome or gene transcriptome of an individual cell, thereby obtaining multiple omics data, including gene, transcript, and protein information. Subsequently, it elucidates the relationships between cell population variations and differences in gene expression [14]. Single-cell sequencing technology offers significant advantages in analyzing cell-to-cell heterogeneity, identifying rare cell populations, and mapping cell lineages. It empowers researchers to explore cell functions, enabling them to gain deeper insights into cell differentiation processes, lineage relationships, and the trajectories of disease progression trajectories. Moreover, this technology facilitates the discovery of rare cell types, potential biomarkers, and drug targets [15].

The transition journey from CINIII to CSCC is an intricate and multifactorial process, involving a cascade of cellular and molecular events. Deciphering these underlying mechanisms is not only of fundamental scientific interest but also holds the key to devising more efficacious preventive and curative interventions [16]. CSCC is hypothesized to result from the dedifferentiation of cervical epithelial cells triggered by hrHPV infection, with cervical stem cells (CCSCs) playing a pivotal role in the transformation from CINIII to CSCC [17]. Notably, cervical lesions may be spatially heterogeneous, and lesions of different grades, including CINIII and invasive carcinoma, can coexist within the same patient. As the CINIII advance, cervical stem cells experience profound phenotypic metamorphoses. They begin to lose their tightly regulated differentiation blueprints, veering off course and adopting a more aggressive and tumorigenic identity. This transformation is not an isolated cellular event; it is intertwined with changes in the surrounding stromal compartment. The stroma, once a nurturing bed, now becomes an accomplice in the disease process. It secretes a plethora of growth factors, cytokines, and extracellular matrix components that establish a feedback loop with the aberrant stem cells. This crosstalk promotes angiogenesis, ensuring a rich supply of nutrients for the growing tumor mass, and facilitates immune evasion, allowing the cancerous cells to dodge the body’s natural defense mechanisms [18]. Studies have shown that as CINIII towards CSCC, fibroblasts undergo phenotypic changes. They secrete various growth factors, cytokines, and extracellular matrix components that can either promote or inhibit tumorigenesis. For instance, fibroblast-derived factors may enhance epithelial cell proliferation, migration, and survival, facilitating the transition from pre-malignant to malignant states [19]. Moreover, the crosstalk between fibroblasts and immune cells within the cervix further modulates the tumorigenic process. Fibroblasts can influence immune cell recruitment and activation, creating an immunosuppressive or immunostimulatory milieu [20]. Leveraging single-cell sequencing technology, we investigated the alterations in cell types and molecular mechanisms within cervical epithelial cells and their microenvironment during the transformation from CINIII to CSCC. Our aim was to identify non-invasive strategies, such as drug therapies, that could decelerate the progression of CINIII.

## 2. Methods

### 2.1. Study Design and Sample Description

To investigate the cellular and molecular alterations during the transition from CINIII to CSCC, we employed a paired-sample design. A total of 60 patients with hrHPV16 infection were enrolled, from whom we obtained two sets of samples for validation: xenocontrols (30 matched pairs of CINIII and CSCC tissues from different individuals) and self-controls (30 CSCC specimens with adjacent CINIII regions within the same tissue block). Additionally, three pairs of fresh cervical epithelial tissues (CINIII and CSCC from the same patient, constituting spatially co-existing lesions) were specifically collected for single-cell RNA sequencing (scRNA-seq) analysis as the discovery cohort.

For clarity and reproducibility, the key attributes of all cohorts, including sample pairing, tissue processing, and their specific application in each figure, are summarized in Supplementary Table S1. All tissues were obtained from surgical resections at Tianjin Medical University Cancer Institute and Hospital. For scRNA-seq, fresh tissues were immediately immersed in a living cell preservation solution and transported on ice for whole-tissue dissociation into single-cell suspensions on the day of surgery. For validation studies, formalin-fixed paraffin-embedded (FFPE) blocks were used for immunohistochemistry (IHC) and immunofluorescence (IF) staining.

### 2.2. Human Specimens

This study was approved by the Clinical Research Ethics Committee of Tianjin Medical University Cancer Institute (approval number: EK2023094). Informed consent was obtained from all participants prior to sample collection. Clinical specimens, along with case information, were collected from patients infected with hrHPV16 who had CINIII or CSCC. All samples were confirmed to be exclusively positive for hrHPV16 through both clinical genotyping and our in-house HPV sequence alignment analysis; samples co-infected with other hrHPV types or positive for hrHPV types other than 16 were excluded. The exclusion criteria were as follows: (1) patients with severe concomitant systemic disorders; (2) a history of chemotherapy or radiotherapy; (3) any history of malignant tumors other than CINIII or cervical cancer (CC); and (4) patients with presenting evidence of distant metastasis. For validation analyses, two independent tissue-validation strategies were used. First, 30 CSCC specimens containing adjacent CINIII regions within the same tissue block were selected as autologous self-controls, allowing paired comparisons within the same patient and minimizing inter-individual variability. Second, an independent allogeneic validation cohort consisting of 30 CINIII specimens and 30 CSCC specimens from different patients was used to evaluate whether the observed molecular changes were reproducible across individuals. The autologous and allogeneic cohorts were analyzed separately and were not pooled for statistical testing. Moreover, three pairs of fresh cervical epithelial tissues from CINIII and CSCC cases were obtained for subsequent single-cell sequencing analysis.

### 2.3. Construct a Standard Sequencing Library

A total of six cases of CINIII and CSCC epithelial tissues infected with hrHPV16 were selected. Fresh specimens were collected on the day of surgery and immediately placed in a living cell preservation solution. According to the operation manuals of our research group and SEEKGENE Company, one CINIII and one CSCC epithelial tissue were processed into single-cell suspensions. An equal volume of Trypan Blue dye was added to the single-cell suspension for cell counting, ensuring that the concentration of viable cells exceeded 90%. The single-cell transcriptome information was obtained using a single cell 3′ transcriptome kit, which included a chip, oil, gasket, gel microbeads, amplification reagents, library construction reagents, and single cell data analysis software. Leveraging the principle of microfluidic technology, this kit achieved single cell separation and capture via the oil-in-water method. Moreover, nucleic acid-modified Barcoded Beads were used to perform molecular labeling on RNAs from different cell sources, ultimately constructing high-throughput single-cell transcriptome libraries compatible with BDA sequencers. During the single-cell RNA capture and reverse transcription processes, the main peak fragment size of the qualified library ranged from 350 to 750 bp, with no small fragments present. In case small fragments were detected, a second purification was carried out until they were completely removed, as verified by the Agilent 4200 TapeStation (Agilent Technologies, Santa Clara, CA, USA). The library concentration was maintained at no less than 1 ng/μL, measured using a Qubit 4.0 fluorometer (Thermo Fisher Scientific, Waltham, MA, USA). Sequencing was performed on the Illumina (Illumina, San Diego, CA, USA) or BGI high-throughput sequencing platforms. When the final sequencing library was computationally sequenced, it was recommended to add more than 5% balanced libraries. For the present study, all single-cell libraries were sequenced to a minimum depth of 50,000 reads per cell.

### 2.4. Bioinformatics Analysis

After acquiring the raw sequencing data, bioinformatics analysis was conducted using tools provided by SEEKGENE (Beijing, China). The analysis mainly encompassed the following aspects: data quality control, construction of the expression matrix, cell clustering, differential gene analysis, and enrichment analysis. The raw image files obtained from high-throughput sequencing machines were converted into sequenced reads via CASAVA base recognition and stored in FASTQ format. FASTQ is a commonly employed text format for storing biological sequences and their corresponding quality values. In the case of PE150 sequencing, the data obtained had a length of 150 bp at both ends. It was essential to perform quality control on the raw sequencing data, which involved removing low-quality reads, trimming adapters, calculating sequencing error rates, as well as computing Q20, Q30, and GC content. The trim method was utilized to eliminate sequencing adapters and low-quality fragments from the sequencing data to ensure efficient utilization of the sequencing data. All subsequent analyses were based on the clean data. Read1 contained barcode and UMI sequences used for single-cell library construction, whereas read2 represented the insert fragment for the transcriptome. During raw data trimming, fastp software (version 0.23.2) was employed and processed as follows: low-quality reads were truncated using a sliding window approach, with a window size of 4 bases. If the average base quality value within the window was below 10, the reads were truncated starting from that window.

Following quality control, gene expression quantification was carried out by aligning the sequencing data with a reference genome, generating a cell gene expression matrix for subsequent clustering analysis and other downstream analyses. Each read obtained from sequencing was tagged with a barcode and UMI, and reads sharing the same barcode originated from the same cell. During statistical analysis phase, the data were merged to obtain a preliminary expression matrix. This preliminary matrix incorporated both cellular and non-cellular (background) data, which were further filtered to derive the cellular expression matrix. By employing the analysis methods of Cell Ranger and EmptyDrops, the preliminary expression matrix was filtered. Firstly, an expected number of cells (N, with a default value of 3000) was specified, and then the barcodes were sorted in descending order based on their total UMI counts. Two filtering methods were applied: in the first method, the 99th percentile of the initial UMI values was taken as the maximum estimated total UMI count (m), and the barcodes with a UMI count exceeding m/10 were selected as the final captured cells. The second method utilized the expression characteristics of RNA to distinguish cells from backgrounds, enabling the identification of cells with low levels of RNA expression levels. These two methods were combined to obtain cell expression data, which were then merged to yield complete cell expression data for further analysis.

Seurat software (version 4.3.0) was used for clustering and result visualization. Seurat is an R package (version 4.3.0) designed for analyzing single-cell transcriptome data, offering diverse functions such as t-SNE dimensionality reduction analysis, cluster analysis, and marker gene identification. The LogNormalize (version 4.3.0) command was used to normalize gene expression data, and the FindVariable (version 4.3.0) Features command was employed to select 2000 features. Linear transformation was then applied to standardize the data, followed by principal component analysis (PCA) for dimensionality reduction. Subsequently, 15 principal components (PCs)were selected using graph clustering algorithms to cluster the data. Non-linear dimensionality reduction methods like UMAP or t-SNE were utilized to visualize the data. The method provided by the SingleR package (version 2.0.0), based on the reference dataset, was adopted for cell type annotation. Cell-type identities were further validated using canonical marker genes from CellMarker 2.0 and literature-supported cervical single-cell signatures [20,21,22]. The selected markers were required to show cell-type specificity and consistent expression in the corresponding clusters. This approach annotated the identified cells as the cell type with the highest correlation to the reference dataset by computing the orrelation between the single-cell reference expression profile dataset and the cell expression profile to be identified. The identification results of the dataset in the report served as a reference, and the characteristics of the cell population could be described and validated based on existing relevant genes in the existing literature. Seurat’s FindAllMarkers command was used to analyze the differentially expressed genes in each cluster, and these characteristic marker genes were presented in various forms. Taking the graph-based clustering results of the first sample entered in the information collection table as an example, the top 9 genes were selected to observe their expression across different clusters, and violin plots were drawn. Regarding the graphical display of marker gene expression in each cluster, the t-Distributed Neighborhood Embedding (t-SNE) dimensionality reduction algorithm could effectively project large datasets into two or three dimensions, grouping similar cells together. Based on the t-SNE typing results, specific labeling of key marker genes could reveal the expression status of individual marker genes in each cluster. The identified marker genes of each cluster were collectively displayed in a heatmap. The expression level of marker genes within the same cluster was expected to be higher than that in other groups. The log_10_ (UMI+1) value was normalized prior to clustering. In the heatmap, yellow indicated high expression, while purple denoted low expression. Stemness and dynamic trajectory analysis. To quantify the stemness potential of epithelial subsets, we applied the CytoTRACE algorithm to the normalized epithelial cell–matrix, where a lower score indicates higher stemness. To infer directed state transitions, RNA velocity analysis was performed using the scVelo package (version 0.2.5) Spliced and unspliced counts were used to estimate RNA kinetics and calculate a latent time pseudotime, representing the inferred differentiation trajectory. Gene Set Enrichment Analysis (GSEA) was conducted using the clusterProfiler R package (version 4.6.2) against the Hallmark gene sets to characterize fibroblast subpopulations.

### 2.5. Signal Crosstalk Between Cervical Epithelial Cells and Key Cells in the Surrounding Microenvironment in CIN III and CSCC

We primarily utilize the STRING protein interaction database (http://string-db.org) to analyze the protein–protein interaction network of differentially expressed genes within each cluster, taking into account their interaction relationships. The interaction relationships of target gene sets (such as the list of differentially expressed genes in each cluster) are directly extracted from the database for species included in the database to construct a network. For species not included in the database, we initially apply blastx to align the sequences in the target gene set with the protein sequences of the reference species within the STRING database, and then utilize the protein interaction relationships of the aligned reference species to construct an interaction network.

CellphoneDB software (version 3.1.0) is employed to analyze single-cell gene expression. It enables further analysis of the number and expression levels of ligand-receptor pairs in cell pairs, facilitating the construction of interaction network diagrams between different cell types and predicting potential cell–cell communication relationships. The single-cell gene expression matrix is inputted, and the ligand-receptor information contained in CellphoneDB software is used to analyze the expression abundance of ligand-receptor pairs in each cell pair, based on the pattern where one cell expresses receptors while the other expresses ligands. Based on the aforementioned analysis of the expression abundance of ligand-receptor pairs in each cell pair, the number of ligand-receptor pairs expressed in each cell pair can be obtained, which can serve as a preliminary evaluation of the cell–cell communication relationship. To identify biological correlations, the CellphoneDB software is used to compare all cell types in the dataset in pairs, and further analysis is performed on the number of significantly enriched receptor pairs in each cell pair, as well as the number of receptor pairs with a *p*-value less than 0.05 in each cell pair. Based on the number of ligand-receptor pairs mentioned previously, an intercellular interaction network diagram is constructed, which can visually depict the communication relationships between different cell types. Based on cell communication prediction, NicheNet software (version 2.0.4) is utilized to predict ligand target genes. Further analysis of ligand-receptor activity in ligand-receptor signaling cells is carried out based on the expression changes in specific gene sets. NicheNet software is used to rank ligand-receptor activity according to the degree of change in the expression levels of specific gene sets in ligand-receptor signals. The resulting significant interactions (*p* < 0.05) were used to construct global interaction networks and heatmaps. Specific ligand-receptor pairs for key functional categories (e.g., Chemokine, Growth Factor, ECM) were extracted and visualized.

### 2.6. Immunohistochemistry

Immunohistochemical staining protocols were consistent with our previous investigation [23]. For protein-level validation, the same cohort described in ‘Study design and sample description’ was used. This included 30 self-control pairs (CINIII and CSCC regions from the same tissue block) and 30 xenocontrol pairs (individually matched CINIII and CSCC tissues). For self-control validation, the paired CINIII and CSCC regions were sectioned and placed on the same slide for simultaneous staining to minimize batch effects. Primary antibodies used included SOX2 (1:100, Abcam, ab92494, Cambridge, MA, USA), TXNIP (1:200, Abcam, ab188865, Cambridge, MA, USA), and ARL4D (1:200, Abcam, ab169925, Cambridge, MA, USA). After incubation with the primary antibodies, sections were incubated with an HRP-conjugated mouse/rabbit secondary antibody/linker (1:100, Santa Cruz Biotechnology, sc2357, Dallas, TX, USA). Immunoreactivity was visualized using 3,3′-diaminobenzidine (DAB) as the chromogenic substrate, followed by hematoxylin counterstaining. Each immunostaining run incorporated suitable positive and negative controls.

To minimize batch effects in IHC and IF quantification, samples for each marker were processed using the same staining protocol, antibody source, antigen retrieval condition, chromogenic/fluorescent detection system, and imaging settings. For each marker, CINIII and CSCC samples were included in the same staining batch whenever possible. Image acquisition and quantification were performed using identical parameters, and the investigators performing quantification were blinded to group information. For protein level quantification, staining intensity or positive-cell proportions were normalized to the mean value of the CINIII control group, which was set to 1.0. The autologous and allogeneic validation cohorts were analyzed separately to avoid confounding caused by cohort structure or batch variation.

### 2.7. Immunofluorescence Staining and Imaging

Immunofluorescence staining procedures followed those outlined in our previous study [23]. For protein-level validation, the same cohort described in ‘Study design and sample description’ was used. This included 30 self-control pairs (CINIII and CSCC regions from the same tissue block) and 30 xenocontrol pairs (individually matched CINIII and CSSC tissues). For self-control validation, the paired CINIII and CSCC regions were sectioned and placed on the same slide for simultaneous staining to minimize batch effects. Primary antibodies used included CD31 (1:100, Abcam, ab222783, Cambridge, MA, USA), Von Willebrand Factor (0.5 µg/mL, Abcam, ab15580, Cambridge, MA, USA), CD68 (1:100, Abcam, ab213363, Cambridge, MA, USA), CD86 (1 µg/mL, Abcam, ab270719, Cambridge, MA, USA), and Mannose Receptor (1 µg/mL, Abcam, ab300621, Cambridge, MA, USA). Secondary antibodies used were goat anti-rabbit IgG H&L (1:500, Abcam, ab150077, Cambridge, MA, USA). Imaging was conducted using a universal inverted fluorescence microscope (Leica DMI6000B, Leica Microsystems, Wetzlar, Germany), and ImageJ software (version 1.53t) was employed for image analysis and quantification.

The procedures used to minimize batch effects during IHC and IF quantification are described in Section 2.6.

### 2.8. Quantification of IHC and IF Staining

IHC and IF images were quantified using ImageJ software. For IHC images, DAB-positive signals were separated from hematoxylin counterstaining using color deconvolution. For SOX2 staining, because SOX2 is a nuclear transcription factor, quantification was restricted to nuclear DAB-positive staining after background subtraction, while cytoplasmic/background staining was excluded. The relative SOX2 protein level was calculated by normalizing nuclear SOX2 staining intensity to the mean value of the corresponding CINIII control group, which was set to 1.0.

For ARL4D and TXNIP staining, the integrated optical density (IOD) of the DAB-positive signal was measured within the selected tissue region and normalized to the analyzed tissue area. Therefore, ARL4D and TXNIP expression levels were calculated as IOD per unit area. Relative protein levels were obtained by normalizing each value to the mean value of the corresponding CINIII control group, which was set to 1.0. For CD31 and VWF immunofluorescence staining, fluorescence signals were quantified within the selected tissue region. CD31-positive and VWF-positive overlapping signals were identified by thresholding the corresponding fluorescence channels, and the integrated fluorescence density of the overlapping signal was normalized to the analyzed tissue area. The relative CD31^+^VWF^+^ fluorescence signal was calculated by normalizing each value to the mean value of the CINIII control group.

For macrophage-related immunofluorescence staining, including CD68, CD86, and CD206, quantification was performed using a cell-count-based approach rather than fluorescence intensity or signal area. Marker-positive cells and total DAPI-positive nuclei were counted in each field using ImageJ under identical thresholding criteria. The percentage of marker-positive cells was calculated as the number of CD68^+^, CD86^+^, or CD206^+^ cells divided by the total number of DAPI-positive cells in the same field × 100%. For each specimen, at least five randomly selected non-overlapping high-power fields were analyzed, and the average value was used for statistical analysis.

### 2.9. Statistical Analysis

Statistical analyses were performed using R software (version 4.3.2) and GraphPad Prism (version 9.5.1). For scRNA-seq data, preprocessing, normalization, dimensionality reduction, clustering, and visualization were performed using Seurat. Differentially expressed genes between cell clusters or experimental groups were identified using the Wilcoxon rank-sum test implemented in Seurat (version 4.3.0). Multiple-testing correction was performed using Bonferroni correction or the Benjamini–Hochberg false discovery rate method implemented in R package (version 4.3.2) where applicable, and genes with adjusted *p* < 0.05 were considered statistically significant.

For computational analyses of epithelial differentiation and stemness-associated features, CytoTRACE scores (version 0.3.3) were used as a descriptive measure to evaluate relative transcriptional states across epithelial subsets. No formal pairwise statistical testing was performed for CytoTRACE score comparisons, and these results were interpreted as exploratory and descriptive. RNA velocity and latent-time analyses were also used to infer potential dynamic relationships among epithelial states and were interpreted in combination with pseudotime and CytoTRACE analyses.

For pathway enrichment analyses, including GO, KEGG, and GSEA analyses, statistical significance was determined according to the adjusted *p* value or false discovery rate provided by the corresponding R packages. For comparisons of cell proportions between CINIII and CSCC samples, two-sided statistical tests were applied according to the data structure. Paired comparisons between CINIII and CSCC regions from the same tissue block were analyzed using a paired two-tailed Student’s *t*-test or Wilcoxon matched-pairs signed-rank test, depending on data normality. Comparisons between independent CINIII and CSCC samples were analyzed using an unpaired two-tailed Student’s *t*-test or Mann–Whitney U test.

Quantitative IHC and IF data were analyzed using GraphPad Prism. Protein expression levels, staining intensity, positive area, or positive-cell proportions were quantified using ImageJ as described above. Data are presented as mean ± standard deviation unless otherwise stated. For comparisons between two groups, paired or unpaired two-tailed Student’s *t*-tests were used for normally distributed data, and Wilcoxon matched-pairs signed-rank tests or Mann–Whitney U tests were used for non-normally distributed data. For comparisons among multiple groups, one-way ANOVA followed by Tukey’s post hoc test or Kruskal–Wallis test followed by Dunn’s multiple-comparison test was used as appropriate.

For quantitative IHC, IF, and cell-proportion analyses, normality was assessed before statistical testing. All statistical tests were two-sided. A value of *p* < 0.05 was considered statistically significant. Significance levels are shown as ns, not significant; * *p* < 0.05; ** *p* < 0.01; *** *p* < 0.001; and **** *p* < 0.0001. The statistical test used for each quantitative figure panel is indicated in the corresponding figure legend.

## 3. Results

### 3.1. Single-Cell Transcriptome Landscape in CINIII and CSCC

**Figure 1:**
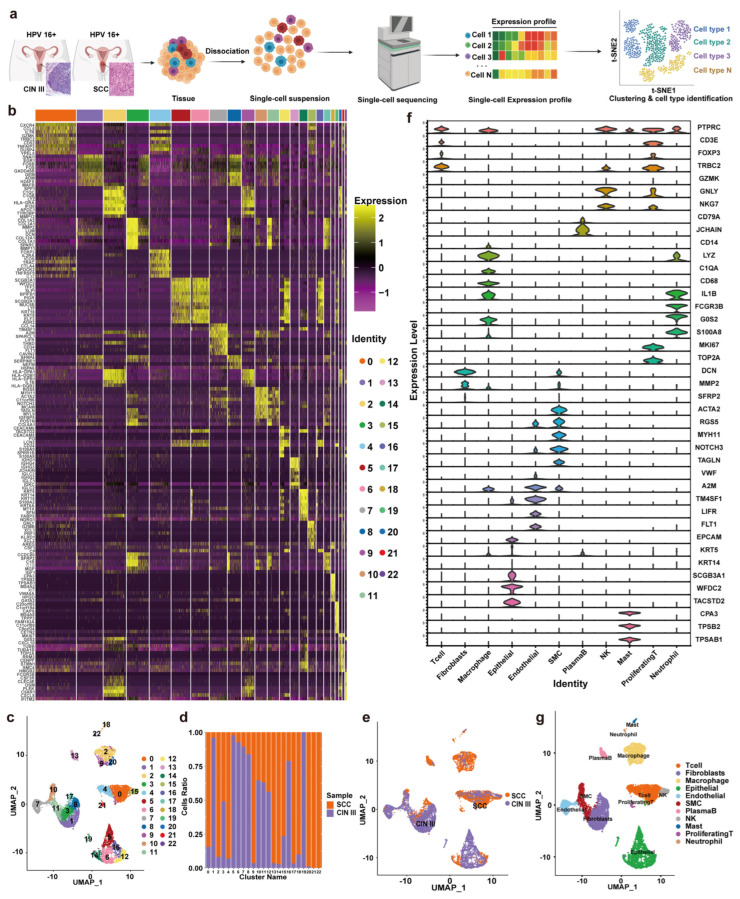
**Single-cell transcriptome landscape in CINIII and CSCC**. (**a**) Single cell sequencing flowchart for CINIII and CSCC, with pathological images showing HE staining of cervical epithelium; Purple indicates CINIII samples, and orange indicates CSCC samples. (**b**) Heatmap of marker gene expression across 23 transcriptionally distinct clusters, re-grouped and color-coded by major cellular categories: Epithelial, Stromal (Fibroblasts), Vascular (Endothelial), and Immune cells; (**c**) UMAP visualization of the 23 clusters, colored by their major cellular categories as in (**b**); (**d**) The proportion of each group of cells in each group; (**e**) UMAP map of single-cell population distribution in CINIII and CSCC; (**f**) Identification of violin plots of gene expression in various cell populations; Each color represents a distinct cell type, including epithelial cells, fibroblasts, endothelial cells, T cells, NK cells, mast cells, proliferating T cells, and neutrophils, as indicated in the legend. (**g**) UMAP diagram for single-cell clustering and identification in CINIII and CSCC.

To delineate the cellular landscape during malignant transition, we performed scRNA-seq on a discovery cohort of three paired fresh tissue samples, each containing spatially co-existing CINIII and CSCC lesions from the same patient (self-controls; see Section 2 and Supplementary Table S1 for cohort details). These tissues were processed into single-cell suspensions for sequencing (Figure 1a). The resulting single-cell transcriptomes were integrated for downstream analysis, with validation performed on 30 paired self-control and 30 xenocontrol FFPE samples, and all cell populations were divided into 0–22 groups based on 9 marker genes for each group, for a total of 23 groups (Figure 1b). Unsupervised clustering of all cells yielded 23 transcriptionally distinct clusters (Figure 1b,c). Based on the expression of canonical marker genes (Supplementary Table S2), we annotated and re-grouped these clusters into four major cellular compartments that encapsulate the functional architecture of the cervical microenvironment: (1) the Epithelial compartment, containing basal, secretory, and stem-like cells; (2) the Vascular compartment, comprising endothelial cells; (3) the Stromal compartment, dominated by fibroblasts and myofibroblasts; and (4) the Immune compartment, including T cells, proliferating T cells, B cells, NK cells, macrophages, mast cells, and neutrophils. To provide a more informative immunological classification, the Immune compartment was further subdivided into lymphoid and myeloid lineages. The lymphoid lineage included T cells, proliferating T cells, B cells, and NK cells, whereas the myeloid lineage included macrophages, mast cells, and neutrophils. This refined classification facilitates the interpretation of lineage-specific immune recruitment and signaling programs during the transition from CINIII to CSCC. Cluster each group of single cells using the UMAP dimensionality reduction algorithm and analyze the proportion of each group of cells in CINIII group and CSCC group (Figure 1c–e). Identify the 0–22 groups of single cells and divide them into T cell group, fibroblast group, macrophage group, epithelial cell group, endothelial cell group, smooth muscle cell group, B cell group, NK cell group, mast cell group, proliferating T cell group, and neutrophil group based on 40 genes (Figure 1f). Cluster and label the identified single-cell populations using UMAP dimensionality reduction algorithm and compare the distribution of CINIII and CSCC single-cell populations (Figure 1g). From the diagram, it is evident that during the transition from CINIII to CSCC, notable alterations take place in various cell types, including cervical epithelial cells, components of the immune microenvironment, elements of the vascular microenvironment, and fibroblasts, along with their respective proportions. Moving forward, we aim to conduct a more in-depth analysis of the single cell panorama of effector cells and effector molecular mechanisms associated with the changes in cervical epithelial cells and the overall cervical microenvironment.

### 3.2. During the Transition from CINIII to CSCC, Cervical Epithelial Cells Gradually Transform into Cervical Cancer Stem Cells, with Sox2 Being a Marker Gene for Cervical Cancer Stem Cells

**Figure 2:**
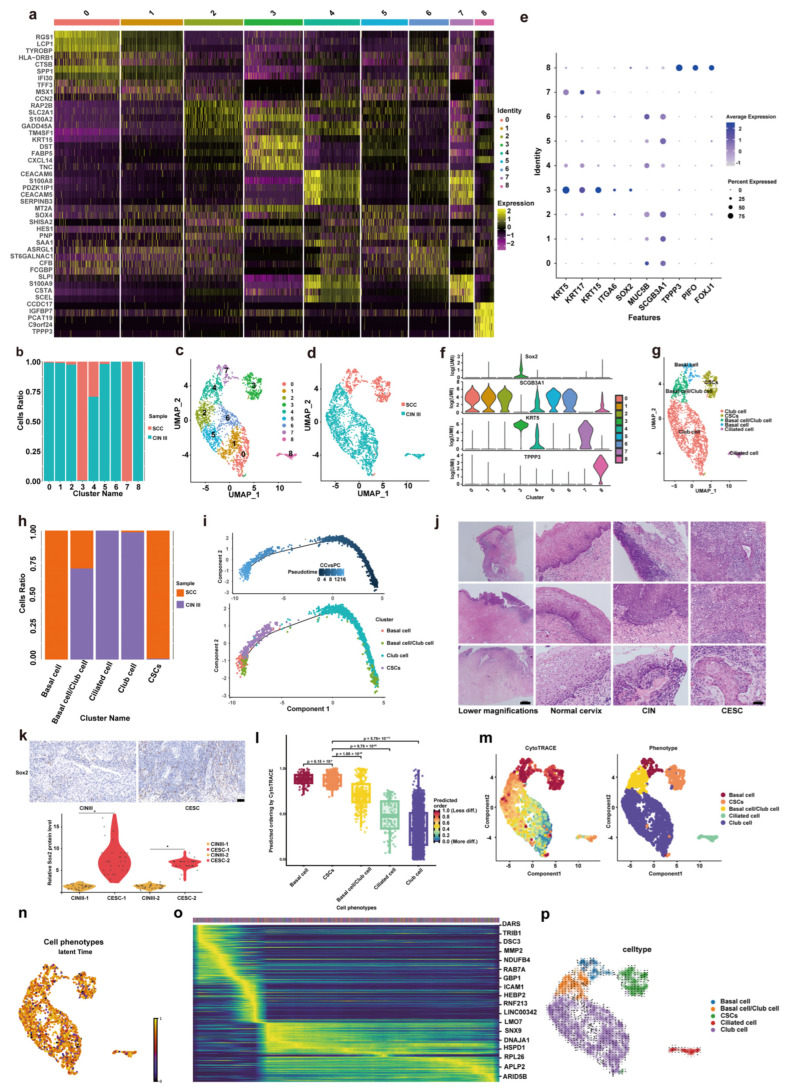
**Cervical epithelial cells gradually transform into cervical cancer stem cells, with Sox2 being a marker gene for cervical cancer stem cells.** (**a**) Heat map of marker gene expression in 9 groups of cells; (**b**) Proportion of each group of cells in each group; (**c**) Represents the UMAP distribution of 9 cell groups; (**d**) UMAP diagram for the distribution of two groups; (**e**) Bubble plot of 10 identified genes expressed in 0–8 cell groups; (**f**) violin plot of key marker genes expressed in 0–8 cell groups; (**g**) UMAP diagram for 9 cell groups, and identification of each cell group; (**h**) Proportion of each type of cell in each group; (**i**) Pseudotime analysis of cervical epithelial cells; (**j**) Representative HE images of CINIII and cervical cancer present simultaneously on a specimen; (**k**) Representative images of Sox2 immunohistochemistry in CINIII and CSCC tissues. The dots represent individual quantified samples. Quantification of Sox2 protein expression was performed based on nuclear DAB-positive staining after background subtraction, while cytoplasmic/background staining was excluded. The relative Sox2 protein level was calculated by normalizing the nuclear Sox2 staining intensity of each sample to the mean value of the CINIII control group, which was set to 1.0. Scale bars, 100 μm. * *p* < 0.05. (**l**) Quantitative assessment of stemness-associated transcriptional features. Box plot showing the distribution of CytoTRACE scores across identified epithelial cell phenotypes. Pairwise comparisons between the SOX2^+^ CSC population and other epithelial phenotypes were performed using the two-sided Wilcoxon rank-sum test (wilcox.test in R), with *p* values indicated in the plot. (**m**) Spatial visualization of the stemness gradient. UMAP projection colored by CytoTRACE score. (**n**) RNA velocity-based latent time analysis of epithelial cells. UMAP embedding colored by latent time inferred from scVelo. (**o**) Genes associated with the dedifferentiation trajectory. He. atmap of genes most correlated with the inferred latent time. Colors indicate scaled gene-expression levels, with yellow representing higher expression and blue/purple representing lower expression. (**p**) Reference epithelial cell states. UMAP visualization of the five major epithelial cell clusters for orientation. For SOX2 immunohistochemistry quantification, paired two-tailed Student’s *t*-test was used for the autologous CINIII–CSCC comparison, and unpaired two-tailed Student’s *t*-test was used for the allogeneic CINIII versus CSCC comparison. *p* < 0.05 was considered statistically significant.

First, we utilized single cell sequencing technology to analyze the differences in cervical epithelial cells. Each group divides all cervical epithelial cell populations into 0–8 groups based on 5 marker genes, for a total of 9 groups (Figure 2a). Cluster each group of single cells using UMAP dimensionality reduction algorithm and analyze the proportion of each group of cells in CINIII group and CSCC group (Figure 2b,c). As shown, the types of cervical epithelial cells in types CSCC were mainly concentrated in groups 3 and 7, whereas the types of cervical epithelial cells types in CIN III are mainly in groups 0, 1, 2, 4, 5, 6, and 8 (Figure 2d), indicating that during the transition from CINIII to CSCC, the types of cervical epithelial cells types tend to become monotonous. Identify the 0–8 groups of single cells and divide all cell groups into basal cell group, goblet cell group, and cervical stem cell group based on 10 genes (Figure 2e,f). Cluster and label the identified single-cell populations using UMAP dimensionality reduction algorithm, and compare the distribution of single-cell populations of cervical epithelial cells in CINIII and CSCC(2g). The main types of cervical epithelial cells in CSCC were basal cells and cervical stem cells, while the cervical epithelial cells in CINIII were mainly goblet cells, basal cells, and ciliated cells (Figure 2h). During the transition from CINIII to CSCC, the epithelial cell composition became more restricted and was accompanied by an enrichment of a SOX2-associated stem-like epithelial population. Thus, SOX2 may represent a putative marker associated with this stem-like epithelial state during CINIII-to-CSCC progression. Pseudotime analysis was conducted on all cervical epithelial cells, and it was found that during the transition from CINIII to CSCC, the degree of cell differentiation gradually shifted towards pluripotency and stemness (Figure 2i). To confirm this sequencing result, we selected 30 cervical specimens with both CINIII and CSCC as autologous controls for verification (Figure 2j), at the same time, we also selected 30 CINIII VS 30 CSCC as allogeneic controls for verification. This transcriptional finding was validated at the protein level using two independent validation cohorts (Supplementary Table S1). Immunohistochemistry confirmed that SOX2 expression was significantly higher in CSCC than in adjacent CINIII regions in the autologous control cohort, and similarly elevated in the allogeneic CSCC cohort compared to independent CINIII tissues (Figure 2k). To comprehensively validate the conversion of epithelial cells into a stem-like state and delineate the underlying dynamics, we performed integrative computational analyses. First, we employed CytoTRACE to quantify differentiation states. CytoTRACE was applied to estimate the differentiation potential of epithelial subpopulations. The SOX2^+^ CSC population showed relatively high CytoTRACE scores, with significantly higher scores than basal/club cells, ciliated cells, and club cells, while the difference between SOX2^+^ CSCs and basal cells did not reach statistical significance, as determined by pairwise Wilcoxon rank-sum tests (Figure 2l). This stemness-associated gradient was also visually apparent across the UMAP embedding (Figure 2m). Second, to explore potential dynamic relationships among epithelial states, we performed RNA velocity analysis. This revealed a latent time distribution across epithelial cells (Figure 2n), which, together with the pseudotime and CytoTRACE analyses, was consistent with a progressive shift toward a stem-like state. Concomitantly, we identified a set of genes whose expression was dynamically regulated along this trajectory (Figure 2o), offering initial mechanistic insights. Collectively, these orthogonal analyses—pseudotime, stemness scoring, and RNA velocity—suggest the gradual acquisition of a stem-cell-like phenotype during the CINIII-to-CSCC transformation.

### 3.3. Analysis of Cervical Endothelial Cell Subpopulations and Marker Genes in CINIII and CSCC

**Figure 3:**
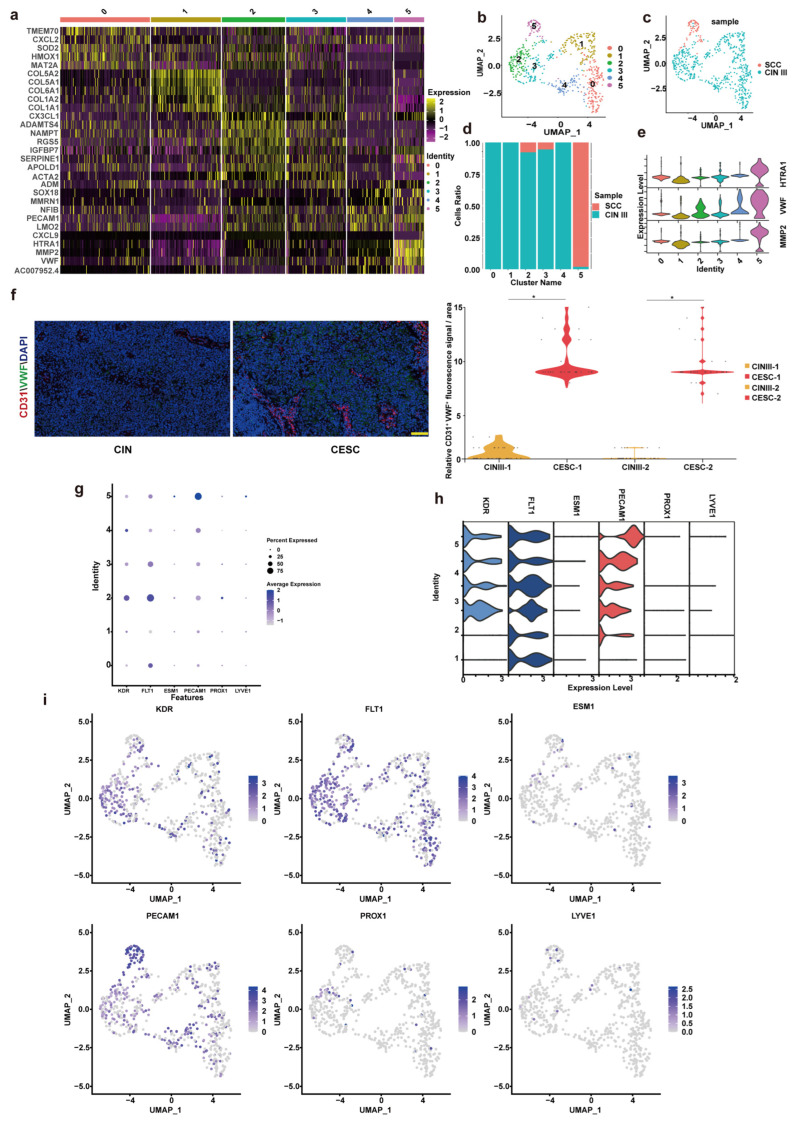
**Analysis of cervical endothelial cell subpopulations and marker genes in CINIII and CSCC.** (**a**) Heatmap of marker gene expression in six cell populations. (**b**) UMAP clustering of the six cell populations. (**c**) Distribution of UMAP clustering within two groups. (**d**) Proportion of each cell population in each group; colors indicate CSCC and CINIII samples. (**e**) Violin plot of marker genes that are highly specifically expressed in the fifth cell population. (**f**) Representative immunofluorescence images of CD31, VWF, and DAPI in CINIII and CSCC tissues. CD31 is shown in red, VWF in green, and nuclei in blue and dots represent individual quantified samples. The CD31^+^VWF^+^ overlapping fluorescence signal was quantified using ImageJ and normalized to the analyzed tissue area. Relative fluorescence levels were calculated by setting the mean value of the CINIII control group to 1.0. Scale bars, 100 μm. * *p* < 0.05. (**g**) Dot plot showing expression of vascular (KDR, FLT1, ESM1, PECAM1) and lymphatic (PROX1, LYVE1) endothelial markers across all subclusters. (**h**) Violin plots comparing the expression distribution of key markers (KDR, PECAM1, PROX1). (**i**) UMAP visualizations of KDR, PECAM1, and PROX1 expression localization. Statistical significance for quantitative IF analysis was determined using a paired two-tailed Student’s *t*-test. *p* < 0.05 was considered statistically significant.

Subsequently, we employed single cell sequencing technology to analyze the differences in the vascular microenvironment during the transition from CINIII to CSCC. As shown in Figure 3, the cervical endothelial cells of CINIII and CSCC were subclustered. These cells were divided into six subpopulations, numbered from 0 to 5 (Figure 3a). Through UMAP analysis and subpopulation proportion analysis, it was found that the cervical endothelial cells of CSCC were mainly in subpopulation 5, while those in CINIII were predominantly in subpopulations 0–4. Subpopulation 5 was mainly characterized by the expression of von Willebrand factor (VWF), matrix metalloproteinase 2 (MMP2), and serine protease 1 (HTRA1) (Figure 3b–e). These characteristics are markers of mature vascular endothelial cells. Subpopulation 5 was mainly characterized by the expression of von Willebrand factor (VWF), matrix metalloproteinase 2 (MMP2), and serine protease 1 (HTRA1) (Figure 3b–e). To definitively validate its endothelial identity and maturation state, we interrogated the expression of canonical lineage markers. Strikingly, cluster 5 exhibited exclusive and high expression of vascular endothelial markers, including KDR (VEGFR2), FLT1 (VEGFR1), ESM1, and PECAM1 (CD31), while showing no expression of lymphatic endothelial markers PROX1 and LYVE1 (Figure 3g,h). This comprehensive molecular signature unambiguously identifies cluster 5 as a population of differentiated, mature vascular endothelial cells. In summary, following the transformation from CINIII to CSCC, the types of cervical endothelial cells differentiate into a single, more mature endothelial cell type. In summary, following the transformation from CINIII to CSCC, the types of cervical endothelial cells differentiate into a single, more mature endothelial cell type. Immunofluorescence was employed to co-stain CD31 and VWF on 30 pairs of autologous control and 30 pairs of allogeneic control cervical epithelial specimens. Through meticulous statistical analysis, a significant increase in the expression of VWF in cervical endothelial cells was observed during the transition from CINIII to CSCC. CINIII-1 and CSCC-1 represent autologous controls, and CINIII-2 and CSCC-2 represent allogeneic controls (Figure 3f).

### 3.4. Analysis of Cervical Fibroblast Subpopulations and Marker Genes in CINIII and CSCC

**Figure 4:**
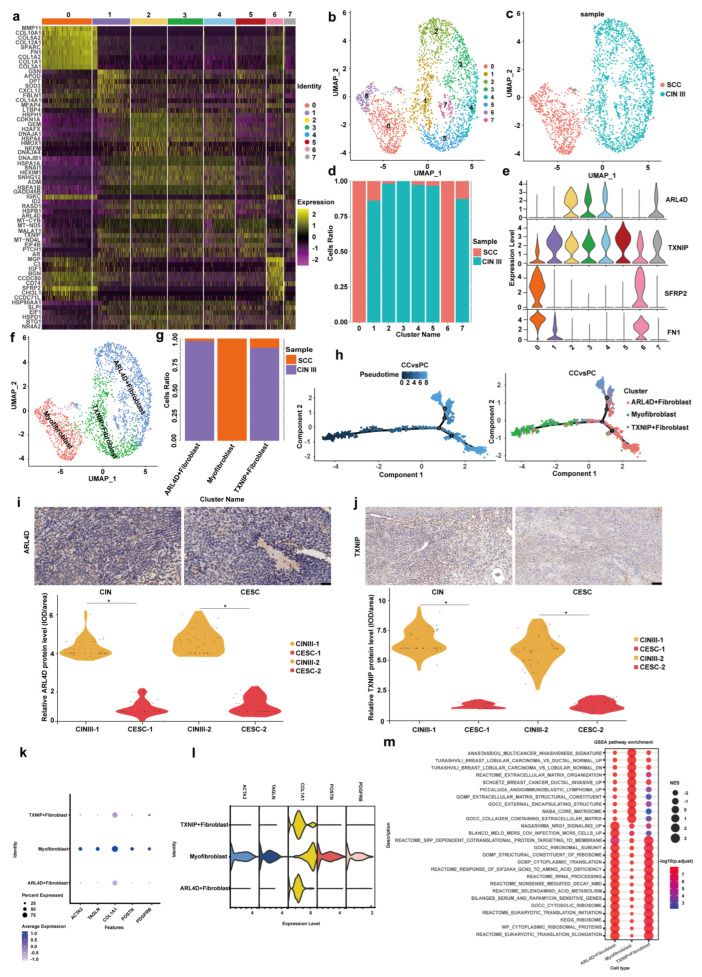
**Analysis of cervical fibroblast subpopulations and marker genes in CINIII and CSCC.** (**a**) Heatmap of marker gene expression in eight cell populations. (**b**) UMAP clustering of the eight cell populations. (**c**) Distribution of UMAP clustering in the two groups. (**d**) Illustrates the proportion of each cell population in each group. (**e**) Violin plot of marker genes that are highly specifically expressed in the eight cell populations. (**f**) UMAP clustering for cell identification. (**g**) Proportion of the identified cells in each group. Subclusters 2, 3, 4, and 7 shared ARL4D expression and were interpreted collectively as ARL4D^+^ fibroblasts in downstream analyses. (**h**) Pseudotime analysis of the fibroblast populations. Each dot represents an individual fibroblast cell, and the black line indicates the inferred developmental trajectory with branch points. In the (**left**) panel, cells are colored according to pseudotime values, showing the predicted progression from early to late fibroblast states. In the (**right**) panel, cells are colored by fibroblast subtype, illustrating the distribution of ARL4D+ fibroblasts, TXNIP+ fibroblasts, and myofibroblasts along the inferred trajectory. (**i**) Representative immunohistochemical staining of ARL4D in CINIII and CSCC tissues, with quantitative analysis based on DAB integrated optical density normalized to tissue area. (**j**) Representative immunohistochemical staining of TXNIP in CINIII and CSCC tissues, with quantitative analysis based on DAB integrated optical density normalized to tissue area. Dots in the quantitative plots represent individual quantified samples/fields. Relative protein levels were calculated by setting the mean value of the CINIII control group to 1.0. Scale bars, 100 μm. * *p* < 0.05. (**k**) Dot plot showing expression of myofibroblast markers (ACTA2, TAGLN, COL1A1, POSTN, PDGFRB) across fibroblast subsets, with highest expression in the terminal Myofibroblast cluster. (**l**) Violin plots quantifying expression distribution of myofibroblast markers, confirming highest levels in the Myofibroblast cluster. (**m**) GSEA dot plot displaying enriched pathways, revealing translation/metabolic programs in TXNIP^+^ fibroblasts and ECM programs in ARL4D^+^ and Myofibroblast subsets. Statistical significance for quantitative IHC/IF analysis was determined using a paired two-tailed Student’s *t*-test. *p* < 0.05 was considered statistically significant.

Fibroblasts, particularly cancer-associated fibroblasts (CAFs), play a pivotal role in tumor microenvironment remodeling by secreting extracellular matrix components, growth factors, and cytokines that drive tumor progression, metastasis, and therapeutic resistance. Therefore, we utilized single cell sequencing technology to analyze the phenotypic changes in fibroblasts during the transition from CINIII to CSCC. As shown in Figure 4, the cervical fibroblasts from CINIII and CSCC were subclustered into eight subpopulations, numbered from 0 to 7 (Figure 4a). Through UMAP analysis and subpopulation proportion analysis, it was revealed that the cervical fibroblasts of CSCC were mainly located in subpopulations 0 and 6, while those in CINIII were predominantly in subpopulations 1, 2, 3, 4, 5, and 7. Subpopulations 0 and 6 were myofibroblasts; subpopulations 1 and 5 were fibroblasts positive for thioredoxin interacting protein (TXNIP); and subpopulations 2, 3, 4, and 7 were fibroblasts positive for ADP-ribosylation factor 4D (ARL4D) (Figure 4b–g). Based on marker-gene expression, subpopulations 0 and 6 were annotated as myofibroblasts, subpopulations 1 and 5 were annotated as TXNIP^+^ fibroblasts, and subpopulations 2, 3, 4, and 7 showed shared ARL4D expression and were therefore annotated collectively as ARL4D^+^ fibroblasts (Figure 4b–g). Notably, although subpopulations 2, 3, and 4 were separated by unsupervised clustering at the selected resolution, they exhibited highly similar marker-gene expression patterns and comparable distribution profiles in downstream analyses. Therefore, we interpreted these subpopulations as closely related transcriptional subclusters within the ARL4D^+^ fibroblast state, rather than as functionally distinct fibroblast categories. Pseudotime analysis suggested a potential phenotypic continuum from TXNIP^+^ and ARL4D^+^ fibroblast states toward myofibroblast-enriched states during the progression from CINIII to CSCC (Figure 4h). Likewise, we utilized immunohistochemistry to stain TXNIP and ARL4D in 30 pairs of autologous control and 30 pairs of allogeneic control cervical epithelial specimens. Upon statistical analysis, it was demonstrated that TXNIP staining in CSCC showed a marked reduction in nuclear localization, whereas cytoplasmic staining was relatively retained. Therefore, the apparent reduction in TXNIP in CSCC mainly reflected loss of nuclear TXNIP signal rather than uniform loss of total cellular staining. In contrast, ARL4D staining was reduced during the progression from CINIII to CSCC. CINIII-1 and CSCC-1 represent autologous controls, and CINIII-2 and CSCC-2 represent allogeneic controls. (Figure 4i–j). In summary, during the progression from CINIII to CSCC, TXNIP^+^ and ARL4D^+^ fibroblast-associated features were reduced, whereas myofibroblast-associated features became more prominent. The roles of TXNIP and ARL4D functions in fibroblasts in preventing the transformation from CINIII to CSCC merit further exploration.

To consolidate the identity of these subsets and decipher their functional states, we performed complementary analyses. Examination of canonical myofibroblast markers (ACTA2, TAGLN, COL1A1, POSTN, PDGFRB) confirmed that the terminal cluster uniquely exhibited high expression of this comprehensive panel compared to the other subsets (Figure 4k,l), providing robust evidence to solidify its annotation as activated myofibroblasts. Gene Set Enrichment Analysis (GSEA) further revealed distinct functional programs: the TXNIP^+^ subset was significantly enriched for pathways related to cytoplasmic translation, ribosomal biogenesis, and cellular metabolism. In contrast, both the ARL4D^+^ subset and the myofibroblast cluster were robustly enriched for pathways governing extracellular matrix (ECM) organization and collagen metabolism, with the strongest enrichment signal observed in the myofibroblasts (Figure 4m). Together, these results support a fibroblast-state continuum from a metabolically active TXNIP^+^ state to an ARL4D^+^ matrix-associated state and finally to a myofibroblast-enriched matrix-remodeling state.

### 3.5. Differences in the Immune Microenvironment Between CINIII and CSCC

**Figure 5:**
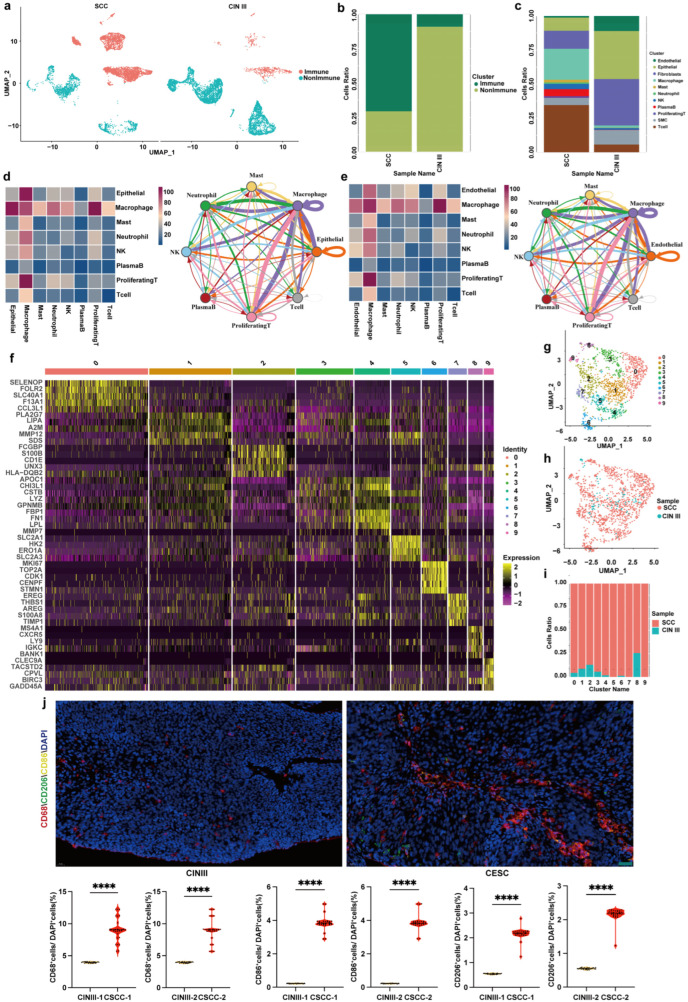
**Differences in the immune microenvironment between CINIII and CSCC.** (**a**) UMAP clustering distribution of immune and non-immune cells in the two groups. (**b**) Proportion of immune and non-immune cells in each group. (**c**) Proportion of various types of immune cells and epithelial cells in each group. (**d**) Analysis of the interaction between various types of immune cells and cervical epithelial cells. (**e**) Analysis of the interaction between various types of immune cells and cervical vascular endothelial cells. (**f**) Heatmap of marker gene expression in ten cell populations. (**g**) UMAP clustering of the ten cell populations. (**h**) Distribution of UMAP clustering in the two groups. (**i**) Illustrates the proportion of each cell population in each group. (**j**) Representative immunofluorescence staining and cell-count-based quantification of CD68, CD86, CD206, and DAPI in CINIII and CSCC tissues. CD68 is shown in red, CD206 in green, CD86 in yellow, and nuclei in blue. Quantification was performed by counting CD68^+^, CD86^+^, or CD206^+^ cells and normalizing them to the total number of DAPI-positive cells in the same field, rather than by fluorescence intensity or signal area. At least five randomly selected non-overlapping fields were analyzed for each specimen. Scale bars, 100 μm. Asterisks in panel (**j**) indicate statistically significant differences between the indicated CINIII and CSCC groups; *p* < 0.05 and **** *p* < 0.0001. Statistical significance for comparisons of immune-cell proportions and quantitative IF results was determined using the Wilcoxon rank-sum test or paired two-tailed Student’s *t*-test, as appropriate. *p* < 0.05 was considered statistically significant.

The cervical cancer immune microenvironment is characterized by a complex interplay of immunosuppressive cell populations and inhibitory cytokines, often driven by HPV oncoproteins that downregulate antigen presentation and promote immune evasion. Thus, we utilized single cell sequencing techniques to elucidate the changes in the immune microenvironment during the progression from CINIII to CSCC. As shown in Figure 5, a comparison was made between the immune and non-immune cells in CINIII and CSCC (Figure 5a). During the transformation from CINIII to CSCC, the proportion of immune cells increased, while that of non-immune cells decreased. Among them, the proportions of macrophages and T cells rose (Figure 5b,c). An analysis of the interaction between all immune cells and cervical epithelial cells revealed that the interaction between macrophages and cervical epithelial cells was the most prominent, and the analysis of endothelial cells yielded comparable results. (Figure 5d,e). The macrophages in the cervical tissues of CINIII and CSCC were subclustered into ten subpopulations, numbered from 0 to 9 (Figure 5f). Through UMAP analysis and subpopulation proportion analysis, it was found that the number of macrophages in the cervix of CSCC increased, and new subtypes emerged (Figure 5g–i). In cervical cancer, tumor-associated macrophages (TAMs) predominantly exhibit an M2-like immunosuppressive phenotype, promoting immune evasion through secretion of anti-inflammatory cytokines. As evidenced by our findings, during the transition from CINIII to CSCC, there is a prevalent increase in both M1-phenotype macrophages and M2-phenotype macrophages. Subsequently, immunofluorescence co-staining for CD68, CD86, CD206, and DAPI was performed in 30 pairs of autologous control specimens and 30 pairs of allogeneic control specimens. CD68 was used as a pan-macrophage marker, CD86 as an M1-like macrophage-associated marker, and CD206 as an M2-like macrophage-associated marker. In the revised Figure 5j, quantification was performed using a cell-count-based approach rather than fluorescence intensity or signal area. Specifically, CD68^+^, CD86^+^, and CD206^+^ cells were counted and normalized to the total number of DAPI-positive cells in the same fields. The percentages of CD68^+^, CD86^+^, and CD206^+^ cells among total DAPI-positive cells were increased in CSCC compared with CINIII, indicating enhanced macrophage infiltration and altered macrophage-associated phenotypes during malignant progression. CINIII-1 and CSCC-1 represent autologous controls, whereas CINIII-2 and CSCC-2 represent allogeneic controls (Figure 5j).

### 3.6. Cell–Cell Communication Analysis Reveals Enhanced Stromal-Epithelial-Immune Crosstalk During Malignant Progression

**Figure 6:**
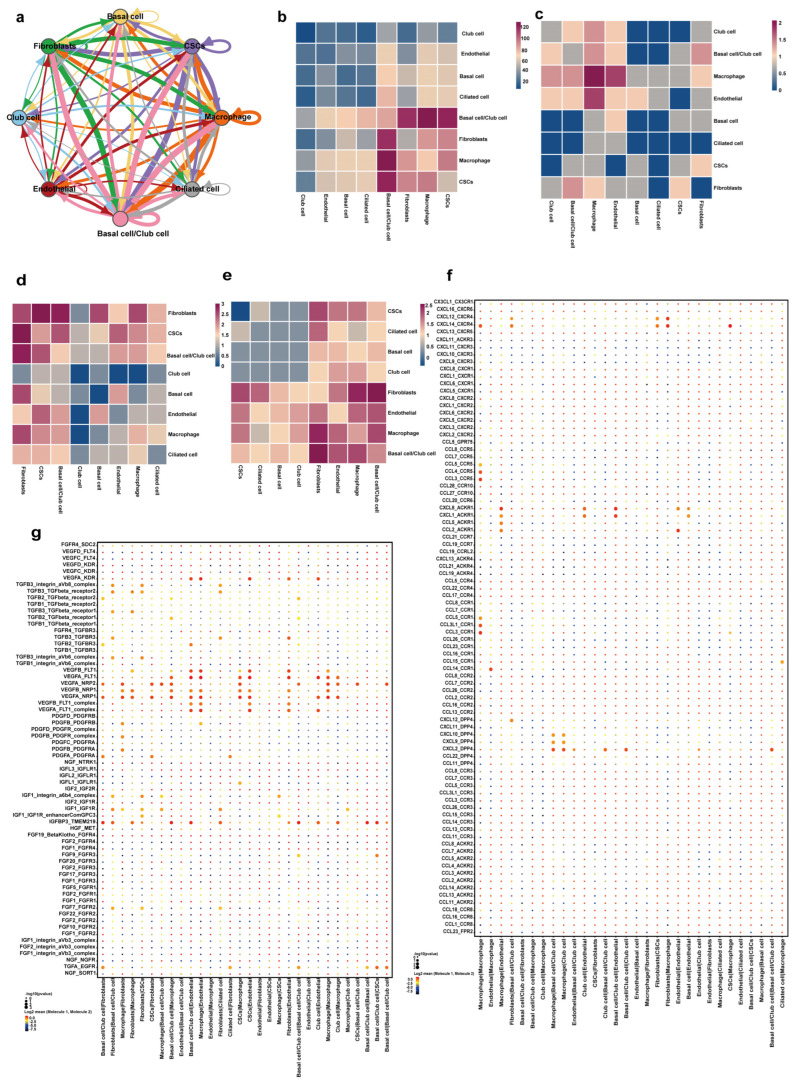
**Cell–cell communication landscape during malignant progression from CINIII to CSCC.** (**a**) Network diagram of predicted ligand-receptor interactions, showing macrophages and fibroblasts as central hubs. (**b**) Heatmap of overall interaction strength between all sender and receiver cell types. (**c**) Heatmap depicting interaction strength specifically for chemokine signaling. (**d**) Heatmap depicting interaction strength specifically for extracellular matrix (ECM)-related interactions. (**e**) Heatmap depicting interaction strength specifically for growth factor signaling. (**f**) Dot plot of the top 30 most significant ligand-receptor pairs in chemokine signaling. Key interactions, such as CCL2-CCR2, are labeled. (**g**) Dot plot of the top 30 most significant ligand-receptor pairs in growth factor signaling. Key interactions, such as VEGFA-FLT1/KDR, are labeled.

To decipher the potential signaling networks among the identified cell populations, we performed a systematic cell–cell communication analysis using CellPhoneDB. The global interaction network demonstrated extensive connectivity, with macrophages and fibroblasts serving as central communication hubs (Figure 6a). The overall interaction strength heatmap further highlighted the most intensive dialogs, particularly between fibroblasts and basal/Club cells, as well as between macrophages and endothelial cells (Figure 6b).

We next dissected the communication landscape by functional categories. The chemokine signaling module was predominantly active between macrophages, endothelial cells, and Club cells, suggesting an immunomodulatory and recruitment-focused crosstalk (Figure 6c). In contrast, the extracellular matrix (ECM)-related interactions were overwhelmingly strong between fibroblasts and basal/Club cells, underscoring a profound stromal-epithelial dialog likely involved in tissue remodeling (Figure 6d). Notably, the growth factor (GF) signaling network revealed potent angiogenic and proliferative cues, most notably between fibroblasts and endothelial cells (Figure 6e).

To pinpoint the specific molecular drivers, we analyzed the top significantly enriched ligand-receptor pairs. For the macrophage-epithelium axis, we found that macrophage-derived CCL2 and its receptor CCR2 on CSCs/basal cells were among the most significant interactions (Figure 6f, *p* < 0.001). For the fibroblast-endothelial axis critical for angiogenesis, the VEGFA-FLT1/KDR pathway emerged as the top enriched signaling pair, with fibroblasts as the predominant source of VEGFA and endothelial cells expressing the receptors (Figure 6g). These data provide a mechanistic map for the observed cellular changes, directly linking immune and stromal activation to epithelial transformation and vascular maturation.

## 4. Discussion

The transition from cervical intraepithelial neoplasia grade III (CINIII) to cervical squamous cell carcinoma (CSCC) is not merely an epithelial event but a comprehensive reprogramming of the tumor microenvironment. By integrating single-cell transcriptomics with spatial validation, our study delineates a cooperative model wherein concurrent changes across four key compartments—epithelium, stroma, vasculature, and immune infiltrate—orchestrate malignant progression. This model moves beyond cataloging individual alterations to propose how inter-compartmental crosstalk, elucidated by our cell–cell communication analysis, drives the transformation. They disrupt the normal functions of Major histocompatibility complex I (MHCI class), p53, Rb, Notch1, Wnt, MAPK, mTOR, and STAT-related pathways [24]. These pathways are key factors that control normal cell growth, differentiation, and immune function. The increase in telomerase activity is associated with epithelial immortalization and tumorigenesis, and hrHPV-E6 is known to activate telomerase activity in cervical epithelium. Thus, the oncogenes E6 and E7 of the hrHPV genome possess the ability to reprogram the host genome, proteome, and intracellular signaling networks within the cervical epithelial niche to sustain and promote hrHPV persistent carcinogenesis. However, there are substantial differences in the progression rate among CINIII patients. If the progression of CINIII patients can be confirmed, patients with slow progression can receive uterine care under close monitoring, while patients with rapid progression should undergo real-time invasive treatment. It has become an urgent need for CINIII patients to distinguish between rapid and slow progression of CINIII and discover methods to delay the progression of CINIII. The mechanism of cervical epithelial progression to CSCC after hrHPV infection has been extensively investigated [25], but the potential molecular mechanisms between CINIII and CSCC have not been studied. It is of great significance to study the alterations in cell phenotype and molecular mechanism during the transition of CINIII to CSCC to explore approaches to delay the progression of CINIII. Although SOX2 signaling, fibroblast activation, and macrophage infiltration have been described in multiple cancers, the novelty of our study lies in placing these processes within the specific CINIII-to-CSCC transition window. Unlike studies focusing on established invasive tumors, our analysis captures the premalignant-to-invasive boundary in an hrHPV-associated cervical epithelial niche. We show that the emergence of a SOX2-associated stem-like epithelial state, fibroblast remodeling toward a myofibroblast-like phenotype, endothelial adaptation, and macrophage-enriched immune remodeling occur in a coordinated manner during this transition. Therefore, our findings extend beyond general tumor biology by proposing a stage-specific, multicellular cooperative model for CINIII progression to invasive CSCC.

If the lesion is not treated, malignant cervical cells will invade the basement membrane of the cervix and form CSCC Central to the epithelial transformation is the emergence of a stem-like state. Our multi-faceted analysis (pseudotime, CytoTRACE, RNA velocity) converges to demonstrate an active dedifferentiation trajectory toward a SOX2-high phenotype (Figure 2), validated at the protein level. While SOX2 overexpression in CSCC versus normal cervix is known, our study uniquely maps its acquisition during the CINIII-to-CSCC transition. Its expression is associated with cancer progression, stem cell properties, and prognosis. Cervical cancer stem cells during the transition from CINIII to CSCC have not yet been clearly identified and characterized, but future studies on Sox2 as a therapeutic target may provide new insights into treatments to delay the progression of CINIII. Studies have shown that Sox2 expression is higher in cervical cancer compared to normal cervix. For instance, Kim et al. found that OCT4 and SOX2 expression was higher in cervical cancer than normal cervix (both *p* < 0.001). Additionally, Sowmya Dayalan and Vijayashree Raghavan’s research demonstrated that SOX2 showed over-expression in CSCC and CINII/III of the cervix, with a significant *p*-value. CINI showed low expression of Sox2. This indicates that Sox2 expression increases as the lesion progresses from low grade dysplasia to high grade dysplasia and invasive cancer. Our results demonstrated that the expression of Sox2 in CSCC was significantly higher than that in CINIII. Crucially, this epithelial shift does not occur in isolation. Our cell–cell communication analysis reveals that these emerging SOX2-high cells are engaged by macrophages through specific ligand-receptor axes such as CCL2-CCR2 (Figure 6f), positioning immune-stromal crosstalk as a potential modulator of the stem cell niche. Understanding the role of Sox2 in cervical cancer stem cells may provide new directions for treatment of the transition from CINIII to CSCC. Targeting Sox2 or the pathways associated with Sox2 may help in inhibiting the progression of CINIII.

Concomitant with epithelial reprogramming is a coupled stromal-vascular remodeling. Fibroblasts undergo a defined phenotypic continuum, losing TXNIP/ARL4D expression and maturing into activated myofibroblasts enriched in extracellular matrix (ECM) pathways (Figure 4). Mechanistically, these activated fibroblasts serve as key signaling hubs. Our communication data pinpoint them as the predominant source of VEGFA, which strongly engages FLT1/KDR receptors on a converging population of mature endothelial cells marked by high VWF, MMP2, and HTRA1 (Figure 3 and Figure 6g). This direct fibroblast-to-endothelium VEGFA signaling axis provides a functional link between stromal desmoplasia and the observed vascular maturation, ensuring tumor perfusion.

Cancer-associated fibroblasts (CAFs) are important components of the tumor microenvironment, influencing tumor progression and metastasis, and playing a significant role in anticancer treatment and prognosis. CAFs have heterogeneity in phenotype, origin, and function. In breast cancer, pancreatic cancer, and other tumor types, CAFs are common stromal cells. CAFs can be derived from resident fibroblasts in tumor tissues, or from bone marrow or adipose tissue [26]. CAFs perform numerous tasks by interacting with tumor cells, stromal cells, and infiltrating immune cells during tumor progression, including promoting tumor cell proliferation, angiogenesis, extracellular matrix remodeling, coordinating tumor inflammation, maintaining cell stemness, and regulating the immune microenvironment [27]. With the maturation of single-cell sequencing technology, CAFs have been identified in many tumors and have shown important roles. Understanding the role of CAFs and CAFS-related markers in specific tumors can facilitate precise and personalized targeted therapy for specific tumors [28]. Our results showed that TXNIP staining in CSCC was characterized mainly by reduced nuclear localization, whereas cytoplasmic staining was relatively preserved. In contrast, ARL4D staining was reduced during the CINIII-to-CSCC transition. The TXNIP^+^ subset exhibits a biosynthetically active state, enriched for translation and metabolic pathways, which may equip it to adapt to microenvironmental stress. The ARL4D^+^ subset shows an early commitment to matrix-related programs. Ultimately, these subsets contribute to the expansion of the terminal, matrix-remodeling myofibroblasts, which is robustly enriched for ECM organization pathways and serves as the primary effector of stromal remodeling. This functional continuum underscores the heterogeneity and specialized roles of CAFs in driving desmoplasia.

TXNIP, known for its regulatory functions in redox homeostasis, has been postulated to play a modulatory role in the fibroblast—associated tumor microenvironment [29]. Fibroblasts in the vicinity of cervical cancer cells are often reprogrammed, adopting a CAF phenotype. TXNIP might be involved in the metabolic rewiring of these fibroblasts. Given that TXNIP can participate in stress-response and transcription-associated regulatory programs, the loss of nuclear TXNIP localization may indicate altered TXNIP-associated regulatory activity in fibroblast states during progression. However, the current IHC data cannot determine whether this reflects altered nuclear transport, protein redistribution, or changes in TXNIP function, and this requires further mechanistic validation. By interacting with thioredoxin, it can influence the antioxidant capacity within the cells. Aberrant TXNIP expression could disrupt the delicate balance between oxidative stress and antioxidant defenses, potentially leading to a more permissive environment for cancer cell growth, invasion, and metastasis [30]. ARL4D, as a member of the ADP—ribosylation factor family, is mainly associated with vesicular trafficking. In cervical cancer fibroblasts, ARL4D could be crucial for the trafficking of molecules that are essential for cell—cell communication and the delivery of growth factors [31]. It may regulate the secretion of cytokines and chemokines by fibroblasts, which in turn can recruit immune cells or further modulate the behavior of neighboring cancer cells. Since the communication between fibroblasts and cancer cells is bidirectional, any dysregulation of ARL4D—mediated vesicular trafficking might disrupt the normal paracrine signaling loops, contributing to tumor progression. From a therapeutic standpoint, the dual targeting of TXNIP and ARL4D holds great promise [32]. Traditional chemotherapeutic agents often focus on directly killing cancer cells but may overlook the supportive role of fibroblasts. By developing drugs that can modulate the functions of TXNIP and ARL4D, we could potentially disrupt the symbiotic relationship between cancer cells and CAFs. Small—molecule inhibitors designed to correct the abnormal activity of either protein or both in combination might be able to reverse the tumor—promoting phenotype of fibroblasts, rendering the tumor microenvironment less hospitable for cancer growth.

A comprehensive analysis of immune cells within the tumor microenvironment is an essential part of elucidating tumor progression. With the latest advancement of single-cell sequencing technology, the systematic study of the tumor immune microenvironment has been realized, and tumor-associated macrophages (TAMs) are the main components of the tumor microenvironment [21]. TAMs are associated with multiple aspects of cancer biology, such as immunosuppression, angiogenesis, tumor metastasis, and resistance to various anticancer therapies. At the same time, macrophages mediate immune phagocytosis, which can ingest, process, and present antigens to effector T cells to promote killer tumor immunity. Macrophages can directly kill tumor cells and improve the prognosis of some tumor patients. An in-depth study of the heterogeneity of TAMs is crucial for tumor immunotherapy [22]. TAMs play a significant role in the development of tumors. It promotes angiogenesis at the tumor site, provides sufficient nutrients and growth environment for tumor proliferation, and simultaneously secretes and recruits various factors to induce tumor cells to invade surrounding tissues and metastasize from the primary tumor site to distant tissues and organs [33]. Our data reposition macrophages as central communication nodes within the transitioning microenvironment. Beyond a mere increase in number or a shift in polarization markers, the cell–cell communication analysis reveals their intensive crosstalk with both epithelial cells (e.g., via CCL2-CCR2) and endothelial cells. This suggests a role in orchestrating multiple pro-tumorigenic programs—potentially modulating epithelial stemness while supporting vascular and stromal remodeling—thereby integrating the immune response into the core cooperative model of progression. Therapeutically, our cell–cell communication analysis directly informs the development of non-invasive pharmacological strategies. The identification of specific, strong interaction axes—most prominently the CCL2-CCR2 signal between macrophages and the emerging SOX2^+^ stem-like epithelial cells—provides a rational blueprint for disrupting the cooperative microenvironment that drives malignant progression. Targeting this axis, for instance with a CCR2 antagonist, represents a paradigm shift from directly ablating the epithelial lesion to modulating the permissive immune-stromal niche that supports it. This approach exemplifies a potential non-invasive, drug-based intervention aimed at delaying or preventing the transition from CINIII to invasive carcinoma by interrupting a critical molecular dialog identified in our study. It reveals that macrophages engage SOX2-high cervical cancer stem cells (CSCs) through specific axes such as CCL2-CCR2, offering a molecular basis for their potential role in modulating stemness and the inflammatory niche. As the CIN lesions progress towards CSCC, the tumor microenvironment undergoes a significant shift. Chronic inflammation, hypoxia, and the presence of tumor-derived factors gradually drive macrophages to polarize into an alternatively activated, or M2-like, phenotype. M2 macrophages are induced by cytokines like interleukin-4 (IL-4) and interleukin-13 (IL-13). They secrete anti-inflammatory mediators such as interleukin-10 (IL-10) and transforming growth factor-β (TGF-β). IL-10 acts to suppress the activity of antigen-presenting cells (APCs), T cells, and NK cells, effectively weakening the anti-tumor immune response. TGF-β not only promotes extracellular matrix deposition, facilitating tumor invasion, but also plays a role in the differentiation of regulatory T cells (Tregs), which further dampen immune surveillance. Understanding the role of macrophages in the CINIII-to-CSCC transition is of utmost importance for developing preventive and therapeutic strategies.

A major limitation of this study is the relatively small discovery cohort used for scRNA-seq, which included three paired CINIII/CSCC cases. Although we validated selected findings using independent tissue cohorts, the limited number of biological replicates may affect the statistical robustness and generalizability of the single-cell observations. Therefore, the cellular trajectories, inferred transitions, and molecular signatures identified in this study should be interpreted as exploratory and hypothesis-generating, and require further validation in larger, multicenter cohorts.

## 5. Conclusions

In summary, our study proposes that the CINIII-to-CSCC transition is driven by a synergistic interplay across the tumor microenvironment, rather than isolated cellular changes. Our findings indicate that the Sox2 and its associated signaling pathway serve as markers of specific cervical stem cells during the transition of CINIII to CSCC. Further analysis revealed that macrophages potentially engage these SOX2-high CSCs through specific signaling axes such as CCL2-CCR2. To meet the oxygen and nutrient demands of cervical cancer cells, the adaptive transformation of cervical vascular endothelial cells is crucial during the progression from CINIII to CSCC. We have confirmed that, in the transformation from CINIII to CSCC, VWF, MMP2, and HTRA1 exhibit differential expression patterns in vascular endothelial cells. Mechanistically, activated fibroblasts (CAFs) act as key signaling hubs, driving endothelial activation and vascular maturation through potent pathways like VEGFA-FLT1/KDR, which corroborates the observed phenotypic changes. During the transition from CINIII to CSCC, the cellular type alterations and molecular mechanisms involving cancer-associated fibroblasts throughout the transformation process merit in-depth exploration. We demonstrated that fibroblasts positive for TXNIP and ARL4D gradually transform into single myofibroblasts. These transformed fibroblasts concurrently exhibit enhanced signaling capacity, establishing dense ECM and growth factor networks with both epithelial and endothelial cells. Concomitantly, TXNIP-associated nuclear localization and ARL4D-associated fibroblast features were reduced during progression, and the biological roles of TXNIP and ARL4D in cervical fibroblasts warrant further investigation. Our results further verified that, during the transformation from CINIII to CSCC, the proportion of immune cells rises while that of non-immune cells declines, with the proportions of macrophages and T cells increasing notably. Interaction analysis between all immune cells and cervical epithelial cells revealed that the interaction between macrophages and cervical epithelial cells is the most prominent. Our cell–cell communication analysis provides the molecular underpinning for these interactions, for instance, the aforementioned CCL2-CCR2 axis between macrophages and CSCs, systematically clarifying the cooperative mechanisms by which microenvironmental cells drive malignant progression.

While this study delineates a cooperative cellular model for the CINIII-to-CSCC transition, it also opens several avenues for future investigation. First, the functional role of the identified SOX2^+^ stem-like state and its dynamic interaction with the macrophage-derived CCL2-CCR2 axis or the fibroblast-associated TXNIP/ARL4D pathway warrants mechanistic validation using advanced models such as cervical organoids or genetically engineered mice. Second, the prominent cell–cell communication axes, particularly those involving macrophages (e.g., CCL2-CCR2) and fibroblasts (e.g., VEGFA-FLT1/KDR), present direct targets for therapeutic exploration. Testing inhibitors of these pathways in preclinical models could assess their potential to disrupt the pro-tumorigenic microenvironment and halt progression. Third, translating our molecular signatures (SOX2, VWF, TXNIP, ARL4D) into a combined biomarker panel for predicting CINIII progression risk requires validation in large, prospective clinical cohorts. Finally, assessing whether the transition landscape described here in hrHPV16-driven lesions is conserved in cancers caused by other hrHPV genotypes (e.g., HPV18, HPV31) will determine the broader applicability of our findings and guide genotype-specific interventions.

## Data Availability

The scRNA-seq data generated in this study will be deposited in a public repository before publication. Other data relevant to the study are included in the article or Supplementary Materials. Custom analysis scripts are available from the corresponding author upon reasonable request.

## Supplementary Materials

The following supporting information can be downloaded at https://www.mdpi.com/article/10.3390/cancers18101674/s1: Table S1. Overview of patient cohorts, sample pairing, processing, and data application; Table S2. Annotation of the 23 transcriptional clusters identified by single-cell RNA sequencing.

## Abbreviations

The following abbreviations are used in this manuscript:

APCs	Antigen-presenting cells
ARL4D	ADP-ribosylation factor 4D
bFGF	Basic fibroblast growth factor
CAFs	Cancer-associated fibroblasts
CC	Cervical cancer
CCSCs	Cervical stem cells
CSCC	Cervical Squamous cell carcinoma
CIN	Cervical Intraepithelial neoplasia
hrHPV	High-risk Human papillomavirus
HTRA1	Serine protease 1
IL-10	Interleukin-10
IL-13	Interleukin-13
IL-4	Interleukin-4
MHCI Class	Major histocompatibility complex I
MMP2	Matrix metalloproteinase 2
PCs	Principal components
PD-ECGF	Platelet-derived endothelial cell growth factor
Sox2	SRY-box transcription factor 2
TAMs	Tumor-associated macrophages
TGF-β	Transforming growth factor-β
Tregs	Regulatory T cells
t-SNE	t-Distributed Neighborhood Embedding
TXNIP	Thioredoxin interacting protein
VEGF	Vascular endothelial growth factor
VWF	Von willebrand factor
